# BET proteins are a key component of immunoglobulin gene expression

**DOI:** 10.2217/epi-2016-0147

**Published:** 2017-03-21

**Authors:** Jung Min Shim, Jin S Lee, Kirsty E Russell, Coen H Wiegman, Peter J Barnes, David Fear, Ian M Adcock, Andrew L Durham

**Affiliations:** 1 Airways Disease Section, National Heart & Lung Institute, Imperial College London, London, SW7 2AZ, UK; 2 MRC & Asthma UK Centre in Allergic Mechanisms of Asthma, Department of Respiratory Medicine & Allergy, King’s College London, London, WC2R 2LS, UK

**Keywords:** B cells, Brd4, bromo and extraterminal domains, epigenetic gene regulation, histone modifications, immunoglobulin, JQ1, Oct2

## Abstract

**Aim:**

BET proteins have been shown to regulate gene expression including inflammatory genes.

**Methods:**

In order to investigate the role of the BET proteins in immunoglobulin production we treated the human B-cell line CLNH11.4 and primary human B cells and ozone-exposed mice with BET inhibitors (JQ1 or IBET151).

**Results:**

Both proliferation and IgG production were reduced by JQ1 in a concentration-dependent manner. JQ1 significantly reduced immunoglobulin gene transcription. *In vivo* treatment of ozone-exposed mice with the BET inhibitor IBET151 similarly inhibited ozone-induced immunoglobulin production. JQ1 did not reduce the protein levels of Brd4 or Oct2 *per se* but reduced the ability of Brd4 and Oct2 to co-immunoprecipitate and of Oct2 to bind to immunoglobulin gene promoters.

**Conclusion:**

Our results indicate that BET proteins including Brd4 play a crucial role regulation B-cell-specific gene expression and immunoglobulin production.

Antibodies are a key component of the adaptive immune system and have roles in both neutralizing and clearing infections and providing long-term immunity from reinfection [1]. Foreign antibodies are recognized by antigen-specific receptors on the surface of B cells [1]. Recognition of antigens leads to activation of an intracellular cascade and the proliferation and maturation of B cells into either plasma cells, which secrete antibodies with the identical antigen recognition properties as the parent B cell, or memory B cells, which provide long-term immunity [1].

Human immunoglobulins consist of two polypeptide chains: the heavy chain, which determines antibody class, and the light chain. There are five classes of heavy chain (α, δ, ε, γ and μ), encoding IgA, IgD, IgE, IgG and IgM, respectively, and two classes of light chain, κ and λ. [1]. While the light chains are encoded in two separate genes (*IGK* and *IGL*) the heavy chain is encoded in a single locus (*IGH*), with the antibody class determined by rearrangements at the locus; each single cell expressing only a single heavy immunoglobulin chain [2].

Inappropriate activation of the immune system can arise leading to the production of antibodies against noninfectious agents including allergens or the body’s own proteins (autoantibodies). The production of autoantibodies is associated with a number of diseases, including rheumatoid arthritis [3], systemic lupus erythematous [4] and chronic inflammatory diseases such as severe asthma [5] and chronic obstructive pulmonary disease [6]. The mechanism through which allergic or autoantibody production occurs is not well understood although it has both heritable and environmental components such as oxidative stress [6].

Immunoglobulin gene expression is primarily regulated at the promoter region, which contains the highly conserved TATA box. The TATA box acts as a binding site for a number of transcription factors including the TBP and RNA polymerase II [7]. IgG genes also contain a highly conserved octamer sequence (5′-ATTTGCAT-3′) that confers B-cell specificity to the promoter; this sequence is bound by two transcription factors, Oct-1 and the B-cell-specific Oct-2 [8,9] In addition to the promoter, the immunoglobulin genes contain numerous enhancer regions, located both upstream and downstream from the gene that aid transcription [10,11].

Epigenetic mechanisms, the heritable and reversible changes to gene expression that are not encoded in the DNA itself, play crucial roles in the regulation of gene transcription [12]. Mechanisms include DNA modifications, such as DNA methylation and alterations to proteins, such as the histones, including methylation, acetylation and phosphorylation [12]. Histone methylation has been identified during variable diversity and joining domain (VDJ) recombination in B cells [13,14], and histones located at the immunoglobulin genes becoming hyperacetylated during cell differentiation into the B-cell lineage [14,15].

Histone acetylation can be recognized by the bromo and extraterminal (BET) family of proteins including Brd4 [16]. Promoter-bound Brd4 recruits other transcription factors including the transcription elongation factor P-TEFb to resolve transitional pausing and allow transcription to occur [17]. Novel inhibitors of epigenetic mechanisms have been recently developed, including JQ1 as an inhibitor of the BET protein family [18]. JQ1 inhibits the binding of BET proteins to acetylated lysine residues and thereby inhibiting transcription [19]. Although Brd4 has been associated with the *IGH* locus in B-cell lymphomas [20], it is unknown whether Brd4 is important for immunoglobulin production.

We hypothesized that Brd4 and histone acetylation regulate IgG release and demonstrated that JQ1, but not the levorotatory enantiomer JQ1(-), significantly reduced the secretion of IgG proteins from human CLNH11.4 B cells. This was associated with reduced expression of *IGKC* and *IGHG1* and using chromatin immunoprecipitation (ChIP) analysis showed that JQ1 inhibited Oct2 binding to the *IGKC* promoter region. The increased expression of IgG antibodies observed an acute ozone (oxidative stress) exposure murine model of lung inflammation [6] was significantly reduced by IBET151 inhibition. Our results indicate that BET proteins play key role in Oct2 *IGKC* promoter binding and transcriptional activation.

## Methods

### CLNH11.4 human B-cell line

The human B lymphocyte hybridoma (B cell and myeloma) cell line, CLNH11.4 (LGC standards, Teddington, UK), was cultured *in vitro* with Roswell Park Memorial Institute 1640 media in 10% fetal bovine serum (FBS) and l-glutamine. For IgG agglutination and real-time quantitative PCR (RT-qPCR), the cells were serum-starved at 0% FBS with l-glutamine 24 h prior to treatment to synchronize the cells and remove any potential proliferative effect on IgG levels. CLNH11.4 cells produce specific IgG1 (κ) antibodies against human cervical carcinoma.

### Primary human B cells

Primary human B cells were isolated from blood using the human naive B-cell isolation kit II (Miltenyi Biotec, Cologne, Germany). Cells were cultured in Roswell Park Memorial Institute media (Invitrogen, CA, USA), supplemented transferrin (35 μg/ml, Sigma-Aldrich, MO, USA), insulin (5 μg/ml, Sigma-Aldrich), penicillin (100 IU/ml), streptomycin (100 μg/ml), glutamine (2 mM; all ThermoFisher Scientific, MA, USA) and 10% FBS (Hyclone, Perbio Biosciences, Tattenhall, UK), 1 μg/ml anti-CD40 antibody (G28.5, ATCC, VA, USA) and 200 IU/ml of recombinant human IL-4 (R&D System, Abingdon, UK).

Cells were cultured for 7 days before being treated with JQ1 or JQ1(-). After a further 3 days, the cells were isolated and cell cycle was measured using the Annexin Assay or lysed in RLT buffer (Qiagen, Hilden, Germany) for RNA extraction. The concentration of antibodies in the supernatant was quantified by measuring the total protein using the bicinchoninic acid assay (Sigma Aldrich).

Primary B-cell viability was measured using the Annexin V Apoptosis detection kit, following manufacture’s instructions (eBioscience, ThermoFisher Scientific).

### Treatments

JQ1 and its negative levorotatory JQ1(-) were purchased from Tocris Bioscience (Abingdon, UK).

### Cell viability (MTT)

MTT assay was performed to check cell viability as previously described [21].

### Proliferation assay (BrdU)

Cell proliferation was quantified using a BrdU assay kit (Roche, Penzberg, Germany) following the manufacturer’s protocol.

### IgG agglutination assay

The levels of IgG antibodies were measured using the Easy-Titer Human IgG (heavy and light chain) Assay Kit (PierceThermo Scientific, MA, USA). The assay was performed following the manufacturer’s instructions. After 24 h serum starvation supernatant was collected, and antihuman IgG (heavy and light chain) sensitized beads were added. Blocking buffer was then added before the plates were read at 405 nm. The levels of IgG were determined against a human serum IgG standard curve (Sigma-Aldrich).

### RNA isolation

Initially, the cells were lysed in RLT buffer and mRNA was extracted using the RNeasy kit, following manufacturer’s instructions (Qiagen, Manchester, UK). Subsequently cDNA was generated using the High Capacity cDNA kit (Life Technologies, Thermo Fisher Scientific).

### Real-time quantitative PCR

RT-qPCR was performed using SYBR^®^ Green (Qiagen) as a nucleic acid stain in a Corbett Rotor Gene 3000 (Corbett Life Sciences, Qiagen) as detailed by the manufacturer. Initial holding steps were performed at 50°C for 2 min followed by 96°C for 10 min. The following cycling step consisted of a melting step at 95°C for 15 s, subsequent annealing step at 60°C for 30 s and an elongation step at 72°C for 30 s. A total of 50 cycles were performed and a melt curve was initiated at the end of the last cycle. The primers for heavy and light chain IgG1 genes (*IGH1G* and *IGKC*, respectively) are shown in Table 1. cT values were generated using a Rotor Gene 6.1 software, and ΔΔcT values were used in the data analysis. Collected data were normalized to the housekeeping ribosomal 18s (*r18S*) mRNA.

### Western blotting

Proteins were extracted by suspending cell pellets (10^6^ cells/ml, 2500 × *g*, 5 min, 4°C) in radioimmunoprecipitation assay buffer (RIPA; Sigma-Aldrich) with Halt™ Phosphatase Inhibitor Cocktail (Thermo Scientific) and Halt™ Protease Inhibitor Cocktail (Thermo Scientific) as instructed by the manufacturer. The cell debris was discarded using a further centrifugation step (14000 × *g*, 15 min, 4°C), and the protein content was quantified using a Bradford Assay (Bio-Rad Laboratories, CA, USA). Samples were run on NuPAGE 4–12% Bis–Tris gels (Invitrogen) in 3-(N-morpholino)propanesulfonic acid (MOPS) buffer and transferred to membranes using the iBlot system (Invitrogen). The membranes were blocked with 5% milk made in TBS-Tween (150 mM NaCl, 20 mM Tris-HCl, pH 8, 0.05% TWEEN 20) and probed with an anti-BRD4 (Santa Cruz Biotechnology, TX, USA, sc56822, 1:200 dilution), anti-Oct2 (Santa Cruz Biotechnology, sc48772, 1:200 dilution) or anti-β-actin (Abcam, Cambridge, UK, AB6276, 1:20,000 dilution) antibodies. After addition of the appropriate alkaline-phosphatase conjugated secondary antibody (Millipore, MA, USA; AP160A, AP132A, both 1:40,000 dilution) blots were treated with enhanced chemifluoerescent labelling reagent (ECF; GE Healthcare, UK) and visualized using a STORM phosphoimager (GMI, MN, USA). Band intensity was compared using ImageQuant TL (version 7) software (GE Healthcare).

### Immunoprecipitation

About 100 μg of protein was suspended in RIPA buffer (including protease and phosphatase inhibitors) to a final volume of 500 μl. The appropriate antibody was added, and the samples were incubated overnight at 4°C. Magnetic protein A/G beads (Millipore) were added and the samples incubated for a further 2 h, after which the magnetic beads were isolated and washed with RIPA buffer. The bead–antibody–protein complex was denatured at 95°C for 5 min and the isolated protein was run on a western blot as described above.

### Chromatin immunoprecipitation

ChIP was carried out using the EZ-Magna ChIP kit A/G (Millipore) following manufacturer’s instructions. In brief, cells were treated with formaldehyde to cross-link the proteins and DNA. Cells were lysed and sonicated to break up the chromatin before specific proteins were immunoprecipitated overnight using 5 μg the anti-Oct2 antibody (SantaCruz Biotechnology, sc48772), anti-Histone H4 acetylated (BioRad, Kidlington, UK, AHP418), anti-RNA polymerase 2 (Santa-Cruz Biotechnology, sc-899) or negative control isotype antibody (Millipore). The recovered protein/DNA complex was washed, the protein was denatured by proteinase K digestion and the DNA was purified. Finally, the DNA was tested by RT-qPCR, as above, to measure the amount of DNA that was bound to the specific protein. The primers used to measure IGK DNA recovery are listed in Table 1. Both primer pairs are located between the J and C regions of the IGK locus (NCBI Gene ID 3514). Upstream binding site 1 is located 2615 bp downstream of the J region and 71 bp upstream of the IGKC region (sequence position 88857953–88857754) and upstream binding site 2 is located 1931 bp downstream of the J region and 714 upstream of the C region (sequence position 88858674–88858397).

### *In vivo* work

C57BL/6 mice were treated as follows: Group 1 = Air + DMSO/Kleptose (n = 8); Group 2 = Air + IBET151 (n = 8); Group 3 = Ozone + DMSO/Kleptose (n = 8); and Group 4 = Ozone + IBET151 (n = 8). Mice were injected (intraperitoneally) 1 h before ozone exposure. IBET151 was dissolved in 10% (w/v) Kleptose in saline (5:95%) and given at 30 mg/kg. Mice had a single exposure to ozone for 3 h at 3 ppm. After 24 h of exposure the mice were sacrificed; bronchoalveolar lavage fluid (BALF) and serum were collected; and lung tissue was snap frozen in liquid nitrogen.

The levels of IgG antibodies were measured in BALF and serum samples using Easy-Titer Mouse IgG Assay Kit (PierceThermo Scientific). Samples were compared with a standard curve of mouse IgG. Antibody levels were normalized to total protein measured by the Bradford Assay.

Lung samples were homogenized using Precellys ceramic beads and homogenizer (Peqlab, Erlangen, Germany). Subsequently, RNA was isolated and cDNA was generated as described above. Gene expression was measured using Taqman qPCR (Life Technologies), using commercial probes for mouse *Igkv1–117* (Mm01742005_g1) and *Ighg1* (Mm01742100_s1) (Life Technologies) and normalized to *18S* gene expression.

### Statistics

Unless otherwise stated all data are shown as mean and standard error. In order to test statistical significance, a Friedman test (for comparing multiple groups without assumption of normal distribution of data) followed by a Dunns post-test was performed using GraphPad Prism version 5.00 (GraphPad Software, CA, USA). For ChIP analysis a one-tailed Wilcoxon’s signed rank test was used. A p-value of <0.05 was considered significant.

## Results

### JQ1 reduced proliferation & IgG production in CLNH11.4 cells

**Figure 1:**
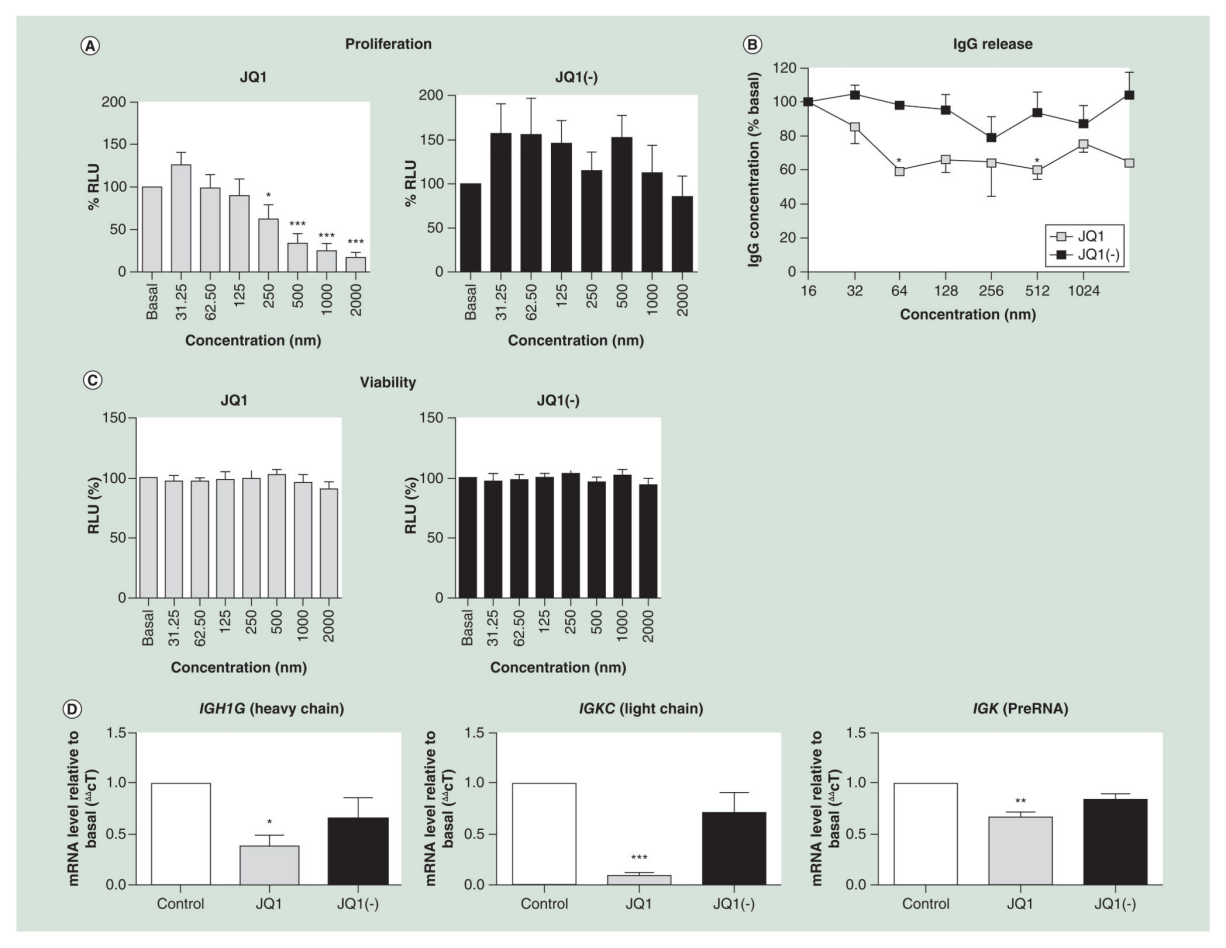
JQ1 significantly reduces B cell proliferation and immunoglobulin production. **(A)** JQ1 but not JQ1(-) significantly reduced cell proliferation, measured by the BrdU assay, at concentrations of 250 nM or higher (n = 6), data shown as percentage relative light units (RLU), relative to the untreated control. **(B)** IgG release, measured by an agglutination assay, was significantly reduced by treatment with JQ1 but not JQ1(-) (n = 5). **(C)** Cell viability, measured by the MTT assay, was not reduced by treatment of the CLNH11.4 cells with either JQ1 or JQ1(-) at any of the concentrations used during this study (n = 7). **(D)** Immunoglobulin encoding mRNA expressions from both the heavy (*IGH1G*) and light (*IGKC*) chains were significantly reduced by treatment with 200 μM of JQ1 but not JQ1(-), as was the preRNA from the κ light chain (n = 3). All data are shown as the mean ± the standard error. *p < 0.05; **p < 0.01; ***p < 0.001 relative to control. RLU: Relative light unit.

JQ1 (IC_50_ = 423 nM) induced a concentration-dependent decrease in cell proliferation which was not observed with the inactive enantiomer JQ1(-) (Figure 1A). The vehicle control (DMSO) had no significant effect on proliferation at any of the concentrations tested (data not shown).

JQ1 also produced a significant reduction in the level of IgG1 released by CLNH11.4 cells over 24 h at concentrations over 64 nM (Figure 1B). No significant reduction in IgG release was detected after treatment with JQ1(-) at any of the concentrations tested (Figure 1B).

No significant changes in viability, as determined by MTT assay, were observed at any of the concentrations tested (Figure 1C).

### JQ1 reduces IgG mRNA expression

There was a significant reduction in both *IGKC* and *IGH1G* mRNA levels after 24 h of treatment with 200 nM of JQ1, compared with control and JQ1(-) (Figure 1C). However, JQ1 did not affect both *IGKC* and *IGH1G* mRNA levels at earlier time points (0, 2 and 6 h; data not shown).

In order to confirm that that the changes to RNA expression occurred prior to splicing, qPCR was carried out against the *IGK* matrix association region, between the *IGK* J and C regions. This exon region of *IGK* RNA contains several important enhancers, putative Oct2 binding sites and the *IGKC* promoter region (ref PMID 2111179). PCR of extracted RNA using primers binding to this region (*IGKC* upstream binding region 2) showed a significant decrease in expression following treatment with JQ1, but not JQ1(-) (Figure 1D).

### JQ1 reduced proliferation & immunoglobulin RNA in primary human B cells

As BET inhibition reduced both B-cell proliferation and immunoglobulin light chain mRNA expression in the CLNH11.4 cell line, we confirmed that JQ1 reduced RNA expression and proliferation in primary human B cells.

**Figure 2:**
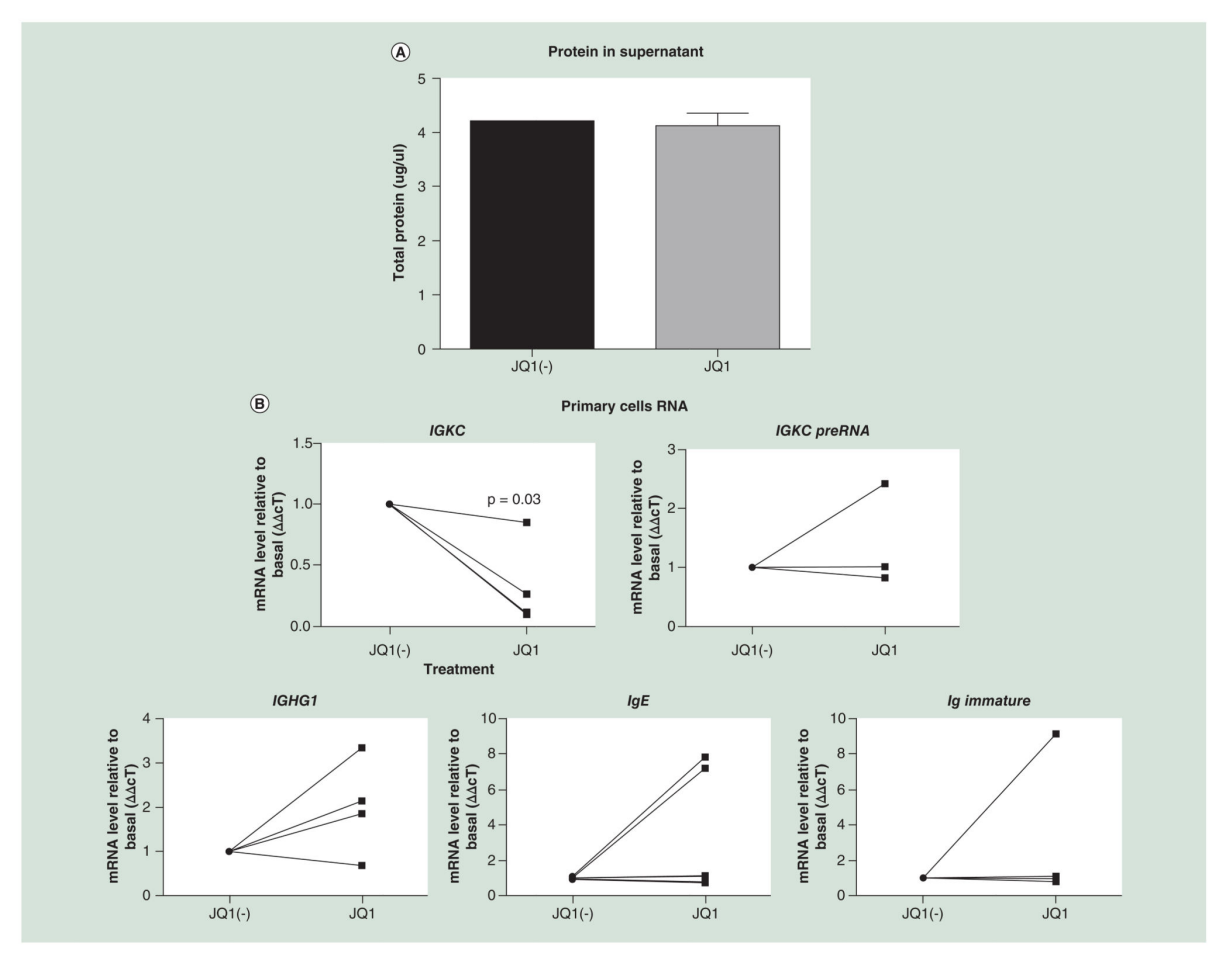
JQ1 inhibits *IGKC* expression in Primary human B cells. Cells were isolated and grown in culture for 7 days, after which they were treated with 500 nM JQ1 or JQ1(-) for a further 3 days. Immunoglobulin production was measured as **(A)** protein in the supernatant, and **(B)** gene expression including light chain mRNA (*IGKC*) and κ light chain PreRNA. Immunoglobulin heavy chain mRNA was also measured against the γ1, ε and immature ε heavy chains (n = 4). The viability of the primary cells was measured by **(C)** Annexin assay **(D)** as was the cell cycle. Data were analyzed using Wilcoxon matched-pairs signed rank test, as the data were nonparametric paired samples.

B cells were grown in culture for 7 days and then treated with JQ1 or JQ1(-) for a further 3 days. After these 3 days, total protein in the supernatant was measured, as a marker of immunoglobulin release; RNA was extracted; and cell cycle/viability was measured. While cell viability was not reduced by JQ1 treatment, there was a significant reduction in *IGKC* and *IGK* preRNA gene expression (Figure 2). This corresponded with a pause of the cell cycle in the G0/G1 phase (Figure 2). Due to these 7 days in culture before JQ1 treatment we were unable to detect a significant reduction in immunoglobulin levels in the supernatant. We did not detect significant changes in heavy chain RNA expression in the primary B cells at the 10-day timepoint, examining the γ, ε or immature ε in heavy chain RNAs.

### BET inhibition reduces BALF antibody levels *in vivo*

In order to confirm the efficacy of BET inhibition at reducing immunoglobulin levels *in vivo*, we examine the ability of the BET inhibitor IBET151 to inhibit antibody production *in vivo*. IBET151 was chosen here, rather than JQ1, owing to our previous experience with IBET151 including dosing experiments. As using IBET151 would not require further dosing experiments, it was therefore chosen for this study, enabling us to again best comply with the NC3R principles. As auto-antibodies have previously been shown to be generated by oxidative stress, including ozone models of chronic obstructive pulmonary disease [6] and can be measured in BALF [22], we used an ozone model to determine if BET inhibition would reduce the induced antibody titers. Using this model also enabled us to look at the effect of BET inhibition on other oxidative stress targets, for example, lung remodeling, which was not included in this manuscript. While potentially suboptimal conducting multiple studies in the same mice enabled us to best comply with the NC3R ‘reduce’ principle.

**Figure 3:**
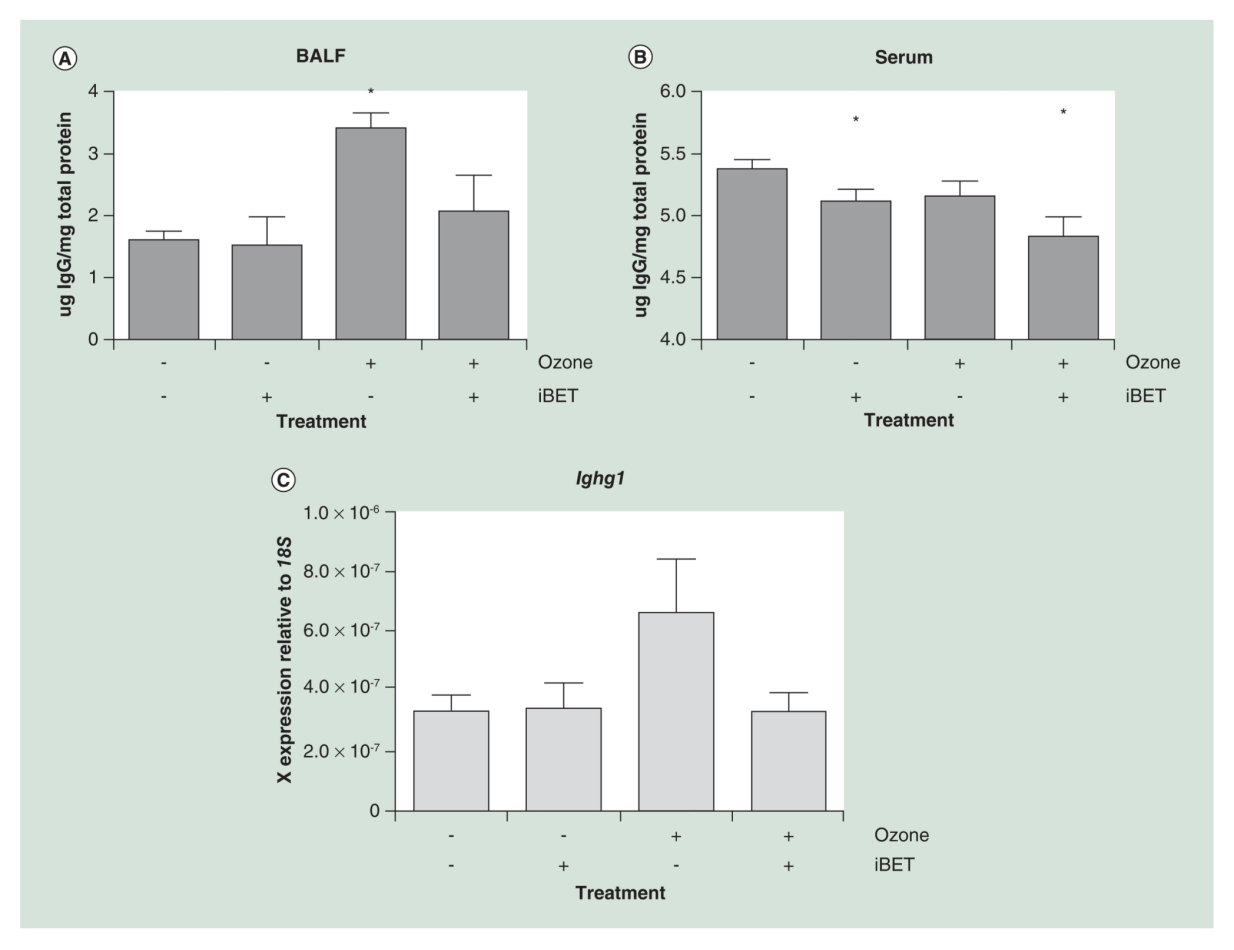
JQ1 prevented ozone induced antibody production *in vivo*. **(A)** Antibody titer in BALF was significantly increased by ozone exposure in acutely exposed mice (n = 8 in each group). This increase was prevented by the treatment of the mice with BET inhibitor IBET151 (30 mg/kg). **(B)** Antibody titer in serum was not significantly altered in serum; however, serum antibody levels were significantly reduced by IBET151 treatment. **(C)** Immunoglobulin gene expression (*Ighg1*) in whole mouse lung tissue was increased following ozone exposure, indicating that the increase in immunoglobulin titer is transcriptionally controlled. All data are shown as the mean ± the standard error. *p < 0.05 relative to air and the placebo group. BALF: Bronchoalveolar lavage fluid; BET: Bromo and extraterminal domain.

Acute ozone exposure significantly increased IgG levels measured in BALF (3.3 ± 0.2 μg IgG/mg total protein) compared with controls (1.58 ± 0.14 μg IgG/mg total protein) (p = 0.003) (Figure 3A). In contrast, IgG levels in the serum were not significantly altered by ozone treatment (Figure 3B).

BET inhibition with IBET151 reduced the IgG levels detected in the BALF (2.0 ± 0.6 μg IgG/mg total protein) in the ozone treated group. Treatment with IBET151 also significantly reduced IgG levels detected in the serum compared with baseline (5.4 ± 0.1 μg IgG/mg total protein), both in the air (5.1 ± 0.1 μg IgG/mg total protein; p = 0.04) and ozone-treated group (4.8 ± 0.2 μg IgG/mg total protein; p = 0.003).

Unfortunately, the numbers of B and plasma cells were not measured in each sample and so it is unknown whether these reductions in gene expression in the whole lung samples reflect reduced cell numbers or changes in gene expression alone.

### BET inhibition reduces IgG gene expression *in vivo*

Gene expression of *Ighg1*, encoding the IgG class antibody heavy chain constant region, was increased following ozone exposure (2.5× increase) (Figure 2C), although this did not reach statistical significance. This increase in *ighg1* gene expression was inhibited in the IBET151 treated group (1.2× increase relative to baseline).

### Treatment with JQ1 does not alter Brd4 or Oct2 protein levels

**Figure 4:**
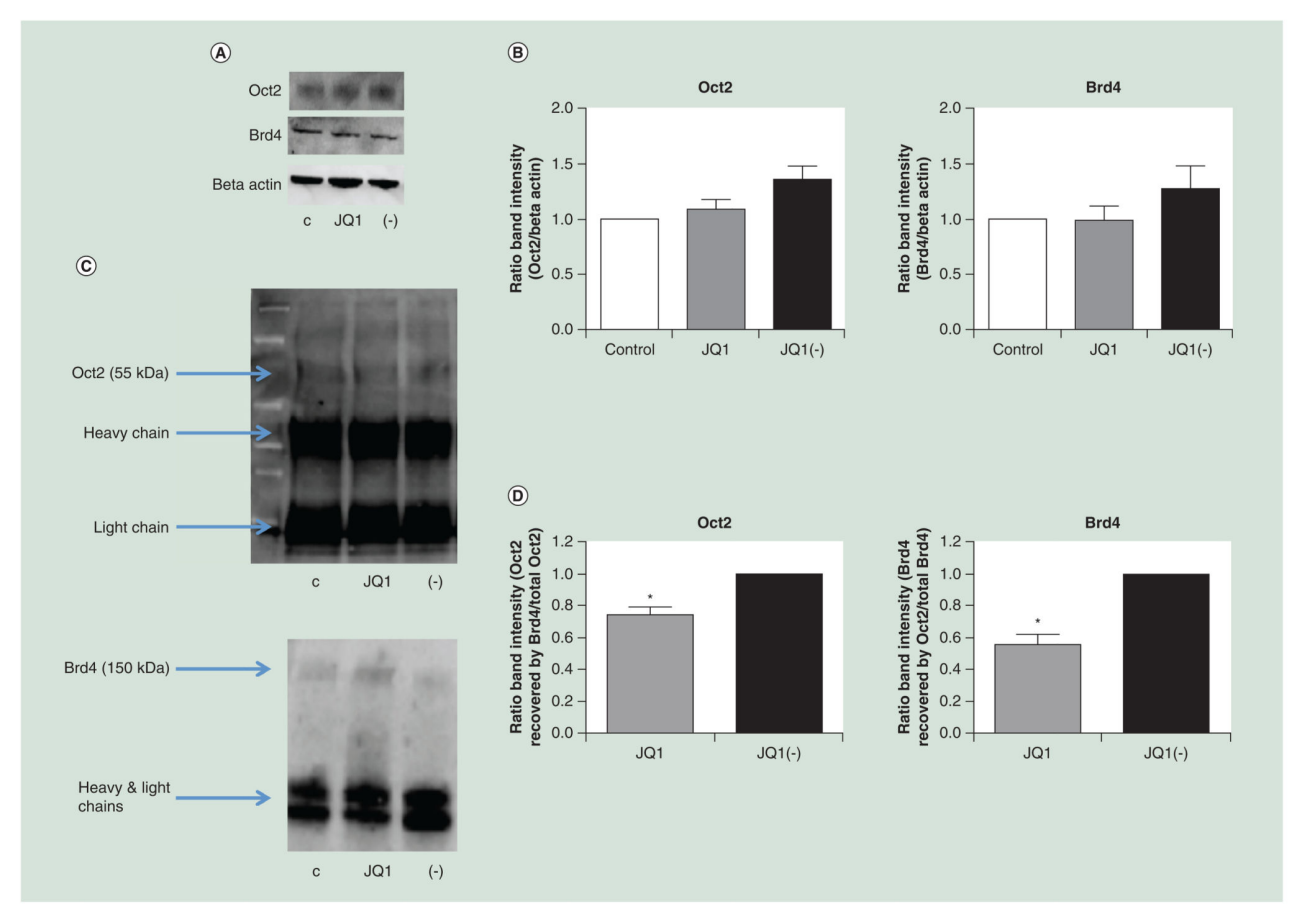
JQ1 reduced Oct2 and Brd4 co-immunoprecipitation. Neither Oct2 nor Brd4 levels were significantly reduced following treatment with JQ1. Proteins were measured by **(A)** western blot and **(B)** quantified (n = 3). Brd4 and Oct2 were both detectable by western blot following immunoprecipitation of either protein. **(C)** A representative western blot of Oct2 and Brd4 protein detected following immunoprecipitation with anti-Brd4 and anti-Oct2 antibodies respectively. **(D)** JQ1, compared with JQ1(-), significantly reduced the amount of Brd4 recovered following Oct2 immunoprecipitation and vice versa (n = 3). C = control, + = JQ1, - = JQ1(-). All data are shown as the mean ± the standard error. *p < 0.05.

Having confirmed the efficacy of BET inhibition *in vivo*, we investigated the mechanisms through which BET inhibition could reduce immunoglobulin mRNA expression. As the Oct2 transcription factor is crucial to B-cell-specific gene expression, its relationship with Brd4 (one of the targets of JQ1) was investigated. CLNH11.4 cells were treated with JQ1 or the enantiomer JQ1(-) for 24 h. After 24 h, the cells were lysed and the protein extracted by whole cell extraction. Protein levels were quantified by western blot analysis (Figure 4A). Treatment of the cells with (200 nM) JQ1 did not alter the expression of Brd4 or Oct2 (Figure 4A & B).

### Brd4 is associated with Oct2

Having confirmed that BET inhibition does not alter Oct2 levels, we investigated whether Brd4 and Oct2 might directly interact. Brd4 and Oct2 were immunoprecipitated from whole cell extracts, and western blot analysis was performed to detect association (Figure 4C). Recovery of Oct2 following Brd4 immunoprecipitation, and *vice versa*, indicates that the proteins are directly bound to each other or are part of the same complex. Treatment with JQ1 (200 nM, 24 h) significantly reduced the co-immunoprecipitation of the proteins compared with JQ1(-) (Figure 4D).

### JQ1 reduces Oct2 *IGKC* promoter association

**Figure 5:**
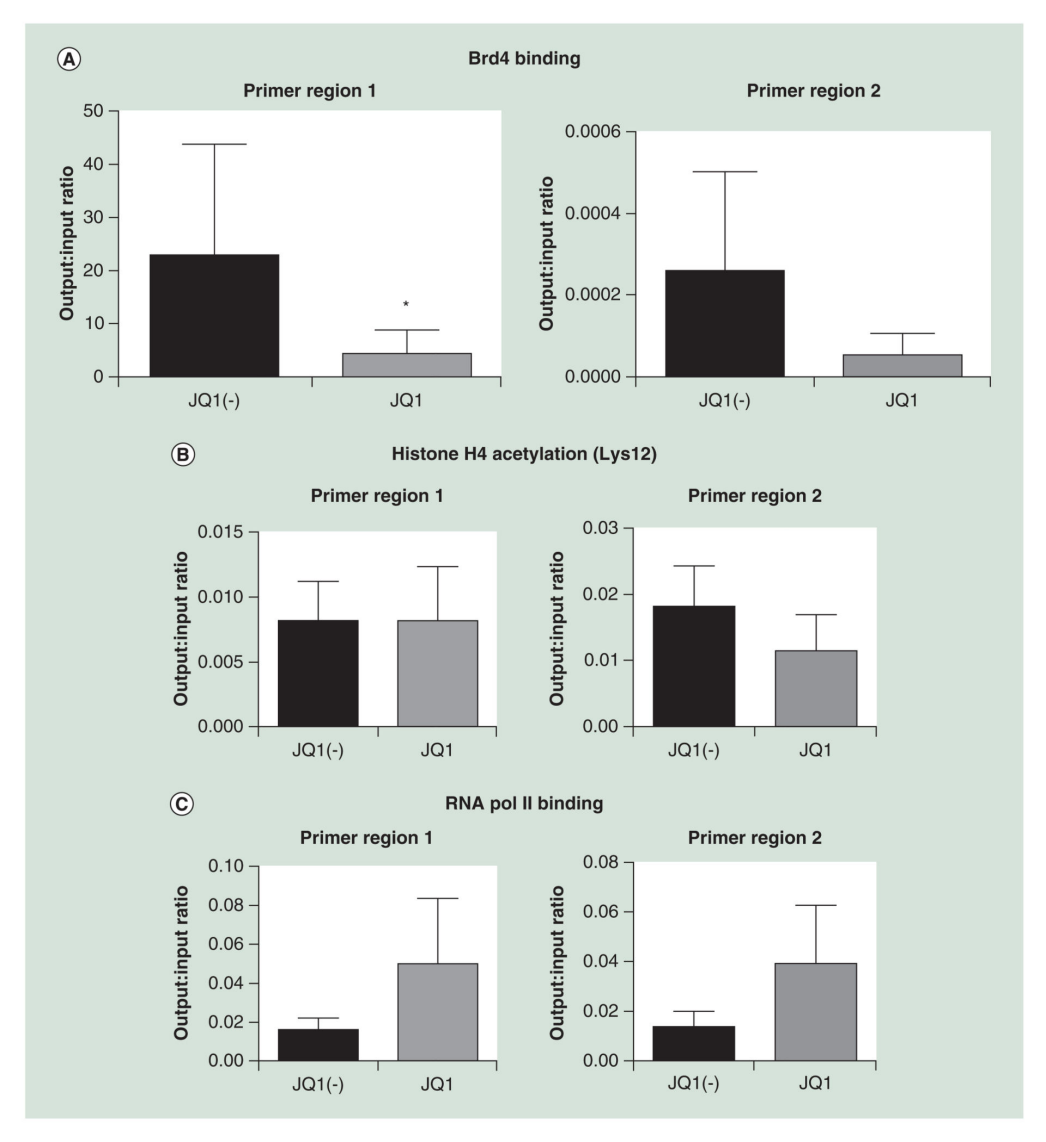
JQ1 significantly reduced Oct2 binding to the *IGK* gene promoter. ChIP was carried out using an **(A)** anti-Oct2 antibody, comparing cells treated with JQ1 versus JQ1(-) controls. PCR of two putative Oct2 binding sites in the *IGK* promoter were carried out (n = 4). In addition, ChIP was carried out using **(B)** antiacetylated histone 4 and **(C)** RNA polymerase 2 antibodies. Data were analyzed using the Wilcoxon matched-pairs signed rank test, as data were nonparametric paired samples. All data are shown as the mean ± the standard error, *p < 0.05. ChIP: Chromatin immunoprecipitation.

It has previously been shown that JQ1 significantly reduces Brd4 binding to the immunoglobulin gene promoter [20]. Since JQ1 significantly reduced Brd4/Oct2 co-precipitation we determined whether the association of Oct2 to the *IGKC* internal promoter/enhancer region was affected by JQ1. The binding of Oct2 to both of its putative binding sites in the *IGK* gene between the J and C regions was reduced by JQ1 (sites 1: 0.41x ± 0.24 vs JQ1(-), p < 0.05 and 2: 0.47× ± 0.38 vs JQ1(-); p-value is not significant) (Figure 5).

In addition, ChIP was carried out against the same regions to look if treatment with JQ1 reduced histone acetylation of RNA polymerase 2 binding. There was no significant change in histone acetylation of the region, confirming the effects of JQ1 on the acetylation reader (Figure 5B). In addition, while not significant, both regions showed an increase in the binding of RNA polymerase 2 (Figure 5C). All antibodies had significantly higher yields of DNA in the output compared with the negative control immunoglobulin (data not shown).

## Discussion

Treating the human B-cell line CLNH11.4 and primary human B cells with the BET inhibitor JQ1, but not its levorotatory enantiomer JQ1(-), significantly reduced proliferation and IgG release without an effect on cell viability. JQ1 also significantly reduced the expression of the IgG encoding *IGKC* and *IGH1G* genes. BET inhibition, by IBET151, also prevented ozone-induced IgG production *in vivo*. JQ1 did not alter the absolute levels of either Brd4 or Oct2 instead reducing their ability to co-immunoprecipitate and Oct2 bind to immunoglobulin genes, indicating that BET proteins play a key role in regulating immunoglobulin gene expression through regulating Oct2 promoter binding.

The BET family of proteins have a role in maintaining gene transcription through cell division, and JQ1 has been previously shown to prevent cell cycle progression from the G_1_ to S phase through an effect on c-Myc [23–25]. The ability of JQ1 to inhibit BET proteins and halt cell cycle progression would explain the loss of proliferation in the B cells *in vitro*.

JQ1 reduced the levels of IgG release from the CLNH11.4 cells into the supernatant by approximately 40% IGH and 90% IGK gene expressions, showing a clear role for the BET proteins in controlling immunoglobulin production at the transcriptional level. Of note is the apparent disparity in the repression of heavy and light chains relative to mRNA levels, especially in the primary B cells. Our data indicate that BET inhibition has a greater impact on the expression of the light chain RNA than the heavy chains. Whether BET proteins do play a more critical role in the light chain, for example, due to the greater complexity of the heavy chain locus, which can undergo processes such as class switching, or whether this reflects experimental complications, such as suboptimal primer design, is unknown but will hopefully be elucidated with further research into the role of the BET proteins in the B-cell gene expression.

The higher level of repression seen in the gene expression compared with immunoglobulin release reflects the fact that CLNH11.4 cells produce the IgG molecules continuously without the need for additional stimulation and therefore will be producing antibodies before the inhibitors take effect. Similarly the primary B cells were grown activated in culture for 7 days before the addition of the JQ1, leading to high background levels of Ig production, making any subsequent JQ1 mediated reduction in immunoglobulin release impossible to detect. The effect of BET inhibition will be only on *de novo* stimulated IgG. The disparity in the data therefore reflects the inability of JQ1 to suppress *IGKC* and *IGH1G* mRNA until 24 h. In contrast, the mouse data show that if BET inhibitors are added before the antibody production is stimulated, both gene and protein expressions are inhibited.

The *in vivo* data showed that BET inhibition reduced in immunoglobulin protein and RNA levels. However, it is unknown whether this is due to simply changes in RNA level or may reflect changes in B-cell numbers, due to the antiproliferative effects of BET inhibition. It would be useful to know the numbers of B lineage cells in the lung tissue, but unfortunately due to practical reasons, we have not been able to assess the lung histology in these samples.

It is interesting to note the apparent discrepancy between inhibition of the heavy and light chains, both *in vitro* and *in vivo*. While both were repressed following JQ1 treatment this is more the case with the light chain, both in the CLNH11.4 cell and the primary B cells. Whether this simply reflects different PCR sensitivity or whether BET proteins play a greater role in regulating the light chain, for example, due to role of enhancer regions in immunoglobulin gene transcription, is beyond the scope of this study but would be worth further study, for example, determining the location of BET protein binding throughout each locus.

The regulation of IgG production by JQ1 at the transcriptional level provides a functional readout for previous data showing that it disrupts Brd4 binding to the *IGH* gene enhancer regions [20] and probably acts through a similar mechanisms to that described in inhibiting inflammatory gene transcription [26–29]. As JQ1 inhibits IgG production at the transcriptional level, and owing to the critical role of Oct2 in B-cell-specific transcription, we investigated the effects of JQ1 on Oct2 expression and function. JQ1 did not affect whole cell Oct2 expression but attenuated the interaction between Oct2 and Brd4. Additionally, treatment with JQ1 significantly reduced Oct2 binding to the immunoglobulin locus (only *IGK* was examined) indicating that the BET family of proteins (Brd4 in particular) is important for the recruitment of Oct2 to the immunoglobulin genes, which fits with previous research showing that acetylation of histones H3 and H4 at the *IGH* enhancer region hs4 is associated with increased Oct2 binding [30].

Brd4 binds to acetylated lysine residues on histones [31] and also other nonhistone proteins [32], through its extraterminal domain [33]. The most studied Brd4’s non-histone targets is the human positive transcription elongation factor b (P-TEFb), which enables Brd4 regulation of RNA polymerase II-dependent gene transcription [17,34]. The increase in DNA recovery from the RNA polymerase ChIP (Figure 5C) would be consistent with transcriptional pause and the interaction of Brd4 with p-TEFb [17]. We therefore believe that JQ1, through binding the acetyl-lysine cavity [19], inhibits BET protein interaction with the acetylated histones. In doing so, it is unable to recruit either Oct2 or p-TEFb leading to reduced transcription and transcriptional pausing.

Other Brd4-associated nonhistone proteins include the RFC-140 subunit of human replication factor C [35], signal-induced proliferation-associated protein 1 (SPA-1) [36], NF-κB [37] and HPV-11 E2 protein [38]. Oct2 has previously been shown to undergo phosphorylation and glycosylation modifications [39], and may therefore also undergo acetylation allowing BET protein binding but whether this is the case is currently unknown. Oct2 protein has been previously shown to interact directly with the TBP [40], which is, in turn, part of the RNA polymerase II transcription initiation complex. Therefore, it is possible that Brd4 and Oct2 do not directly interact but are part of the same transcription complex. While our data indicate that Brd4 directly mediates Oct2 binding to the immunoglobulin promoter region, further research will be required to confirm this hypothesis.

While our research identifies a role for Brd4 in the regulation of immunoglobulin gene expression, this does not preclude a role for the other BET proteins. While JQ1 notably inhibits Brd4, it is a pan-BET inhibitor, including Brd2, Brd3 and T [19]. Brd2 and Brd3 have been shown to play independent roles in several cells of the immune system in both humans and mouse models [27]. Finally, knockdown of Brd2, Brd3 and Brd4 separately in noncancer cell systems has shown that the transcriptional profiles that result are quite independent, and also different from pan-BET inhibitor profiles [41].

## Figures and Tables

**Table 1:** Sequences of PCR primers used in this study.

Primer name	Primer sequence (5′–3′)
**RT-qPCR analysis**	
γ heavy chain (*IGH1G*) forward	GGACCAAGGTGGAAATCAAA
γ heavy chain (*IGH1G*) reverse	GGGAGAGGCTCTTCTGTGTG
κ light chain (*IGKC*) forward	GCTTCTATCCCAGCGACATC
κ light chain (*IGKC*) reverse	TTGGCCTCTCTGGGATAGAA
ε (IgE) forward	CATCGGTCTTCCCC
ε (IgE) reverse	GCCCGTGGCCAGGCAGC
ε (Immature) forward	CTGTCCAGGAACCCGACAGA
ε (Immature) reverse	TGCAGCAGCGGGTCAAG
Ribosomal 18s (r18S) (40)	CTTAGAGGGACAAGTGGCG
Ribosomal 18s (r18S) (40)	ACGCTGAGCCAGTCAGTGTA
**ChIP and preRNA analysis**	
*IGKC* upstream region 1 forward	AAGGTCAGAAAAGCATGCAAAG
*IGKC* upstream region 1 reverse	AATCACAGGGCATGTTAGGG
*IGKC* upstream region 2 forward	TTCCAGTGATCACATTATTTTGC
*IGKC* upstream region 2 reverse	TTTGCTGGGAGAAGTCAACA

ChIP: Chromatin immunoprecipitation; RT-qPCR: Real-time quantitative PCR.

## Summary points

### Aim

- This study was undertaken to examine whether the bromo and extraterminal domain (BET) family of proteins plays a role in regulating immunoglobulin gene expression.

### Experimental procedures

- The B-cell hybridoma cells line CLNH11.4 and primary human B cells were treated with the BET inhibitor JQ1. Ozone-exposed mice were treated with the BET inhibitor IBET151. Subsequently, western blotting and chromatin immunoprecipitation were used to identify the interaction of the transcription factor Oct2, Brd4 and the IGKC gene.

### Results

- BET inhibition led to reduced immunoglobulin production and reduced gene transcription, particularly of the light chain. *In vivo* experiments showed that BET inhibition similarly inhibited immunoglobulin production and gene expression in mice. Subsequent work showed that Brd4 and Oct2 co-immunoprecipitation were blocked by JQ1, which also reduced Oct2 binding to the IGKC gene.

### Conclusion

- We have shown that the BET inhibition is able to reduce proliferation and immunoglobulin gene transcription and production in the human B-cell line CLNH11.4 and *in vivo*. Our work shows evidence that the BET proteins, including Brd4, mediate Oct2 binding to the promoter of the immunoglobulin genes.
